# Effectiveness of Tirobot-assisted vertebroplasty in treating thoracolumbar osteoporotic compression fracture

**DOI:** 10.1186/s13018-021-02211-0

**Published:** 2021-01-19

**Authors:** Boyao Wang, Jiang Cao, Jie Chang, Guoyong Yin, Weihua Cai, Qingqing Li, Zhenfei Huang, Lipeng Yu, Xiaojian Cao

**Affiliations:** Department of Orthopaedics, The First Affiliated Hospital of Nanjing Medical University, 300 Guangzhou Road, Nanjing, 210029 Jiangsu China

**Keywords:** Compression fracture, Robot, Vertebroplasty

## Abstract

**Background:**

Percutaneous kyphoplasty is the main method in the treatment of thoracolumbar osteoporotic compression fractures. However, much radiation exposure during the operation harms the health of surgeons and patients. In addition, the accuracy of this surgery still needs to be improved. This study aimed to assess the radiation exposure and clinical efficacy of Tirobot-assisted vertebroplasty in treating thoracolumbar osteoporotic compression fracture.

**Methods:**

Included in this retrospective cohort study were 60 patients (60–90 years) who had undergone unilateral vertebroplasty for thoracolumbar osteoporotic compression fracture at our hospital between June 2019 and June 2020. All showed no systemic diseases and were assigned to Tirobot group (treated with Tirobot-assisted approach) and control group (treated with traditional approach). Fluoroscopic frequency, operative duration, length of stay (LOS), post-operative complications (cement leakage, infection, and thrombosis), and pre-operative and pre-discharge indexes (VAS score, JOA score, and Cobb’s angle) were compared.

**Results:**

The fluoroscopic frequency (*P* < 0.001) and post-operative complications (*P* = 0.035) in Tirobot group were significantly lower than those in control group. The operative duration and LOS in the Tirobot group were shorter than those in the control group, but the differences were not statistically significant (*P* = 0.183). Pre-discharge VAS score and Cobb’s angle decreased, and JOA increased after surgeries in both groups. These three indexes showed a significant difference after surgery in each group (*P* < 0.001), but not between groups (*P*_VAS_ = 0.175, *P*_Cobb’s_ = 0.585, *P*_JOA_ = 0.448).

**Conclusion:**

The Tirobot-assisted vertebroplasty can reduce surgery-related trauma, post-operative complications, and patients’ and operators’ exposure to radiation. As a safe and effective strategy, this surgery can realize the quick recovery from thoracolumbar osteoporotic compression fracture.

## Introduction

The incidence of thoracolumbar osteoporotic compression fracture keeps increasing in the elderly, often leading to low back pain, kyphosis, difficulty walking, and even neurological dysfunction [1, 2]. Vertebroplasty, one of the traditional surgical methods to treat thoracolumbar fracture, is effective to relieve back pain and bed-rest complication [3–5]. In this surgery, however, both surgeons and patients may face a risk of dermatitis, cataract, and cancer, due to high-radiation exposure from repeated fluoroscopy [6–8]. Therefore, how to reduce the radiation dose of surgeons and patients, while ensuring the curative effect of the operation, is a problem needing urgent resolution.

Robotic surgery system has emerged as a major breakthrough in translational medicine (TM) [9]. As a representative, DaVinci robot-assisted system is being widely used in general surgery and urology, but rarely in orthopedic surgery. In recent years, many studies have reported the good performance of robots in joint surgery, such as total knee arthroplasty (TKA) and total hip arthroplasty (THA) [10, 11]. In addition, Tian et al. practiced Tirobot system in spinal surgery procedures, such as pedicle screw implantation, and achieved good results [12, 13]. To date, however, there are insufficient reports on the application of robotic surgery system in percutaneous vertebroplasty.

In this study, the Tirobot system was used to perform vertebroplasty for osteoporotic thoracolumbar compression fracture. Meanwhile, its clinical efficacy and radiation on surgeons and patients were assessed.

## Materials and methods

### Clinical data

Between June 2019 and June 2020, we recruited 60 patients (27 males and 33 females, ages 61–89 years, mean 70 (66,76) years) receiving unilateral vertebroplasty for thoracolumbar osteoporotic compression fracture. The patients were averaged to Tirobot group (30 cases treated with Tirobot-assisted approach) and control group (30 cases treated with traditional approach). Unilateral percutaneous kyphoplasty (PKP) was performed to treat the lesion at T7-L5. An orthopedic robotic system (Tirobot) was introduced into the surgery in Tirobot group. The procedure was approved by the ethical committee of The First Affiliated Hospital of Nanjing Medical University.

### Orthopedic robotic system

**Fig. 1:**
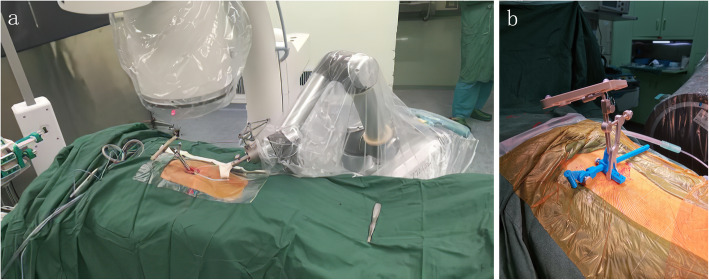
The base of the tracer in Tirobot system. **a** Traditional tracer base. **b** Self-made tracer base

**Fig. 2:**
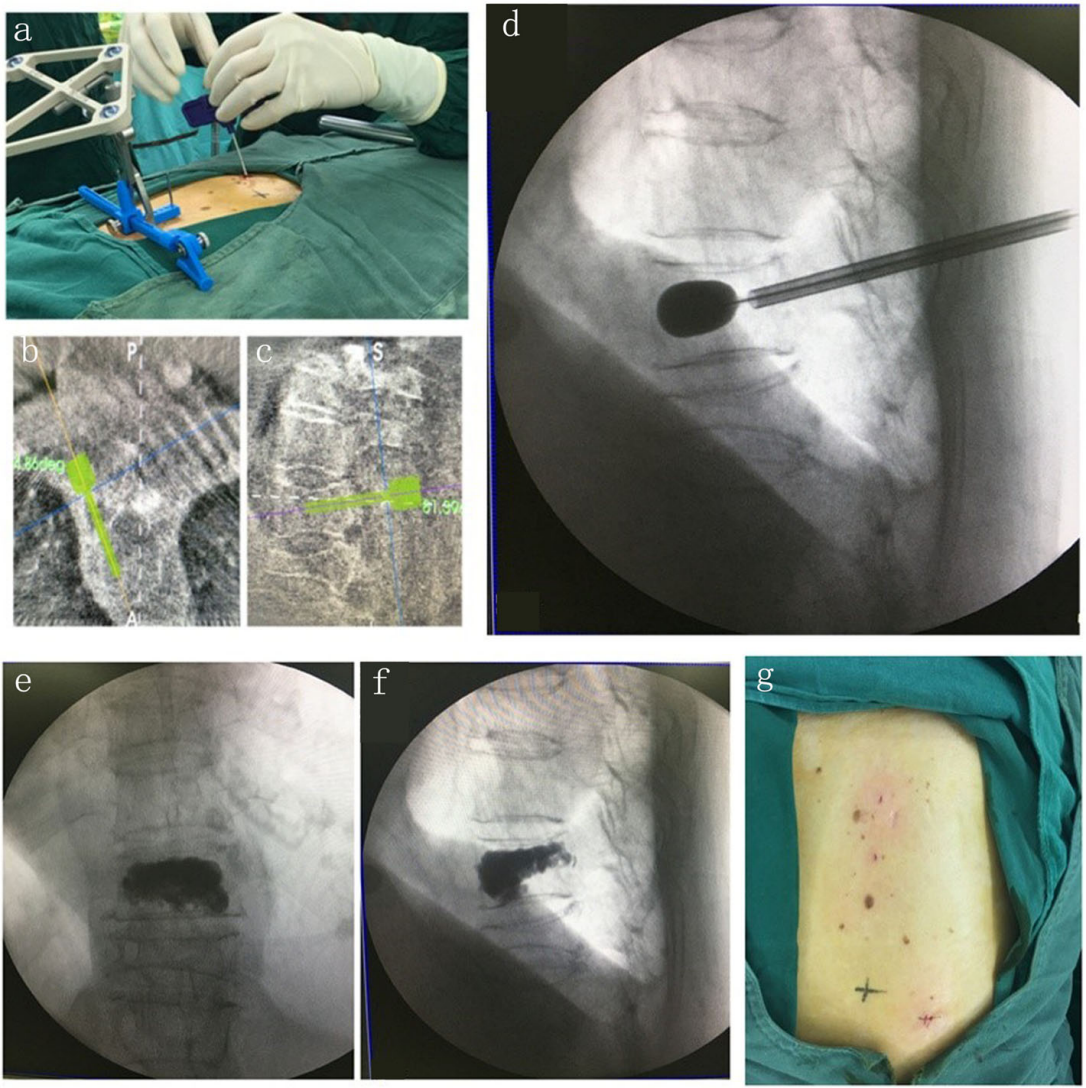
Tirobot system surgical procedure. **a** Cement injection. **b**, **c** Trajectory planning. **d** Balloon placement. **e**, **f** Cement image**. g** Incision

Tirobot is a system consisting of a reference tracker, a robotic arm, and a monitor. During the operation, the manipulator equipped with calibrator was placed at the operation site and aligned with the reference tracker. The C-arm scanner was used to generate three-dimensional images (Fig. 1). The data were loaded via calibrator automatically. The surgeon then planned the trajectory on axial, coronal, and sagittal planes (Fig. 2). The manipulator was adjusted according to the selected trajectory, till displaying the entry point and direction of the implant. A catheter was attached at the end of the robotic arm, allowing surgeons to implant bone cement or screws with the assistance of real-time navigation.

### Surgical procedures

Except for surgical approaches, surgical instruments, and implants were the same in all operations. All interventions were performed by the same group formed by highly experienced surgeons.

### Control group

After general anesthesia, the patient was placed in a prone position. The entry point of the needle was pinpointed by the mobile C-arm. After disinfection and dressing, an incision deep to the fascia was made. With fluoroscopic guidance, a puncture needle was inserted and propelled by a hammer to the posterior vertebral body. A biopsy sheath was inserted through the puncture needle to the anterior third of the vertebral body. After another round of fluoroscopic examination, a reamer was used to make a hole in the vertebra, through which a balloon was placed into to create a void. Then, the bone cement was delivered into the void. After the third round of fluoroscopy, the sheath was removed. The incision was sutured and dressed up with aseptic bandages.

### Tirobot group

After general anesthesia, the patient was fixed in a prone position, followed by disinfection and dressing. K-wire (1.5 mm) was used to connect the tracer and Tirobot system. Under fluoroscopic guidance, the images of lesions were collected. After image registration, the puncture point and angle were designed. Next, through manipulating Tirobot arms, the KMC (Kinetic Medical Co., Ltd) endoscope was targeted to the pedicle axis. An introducer needle (1.6 mm) was inserted through the endoscope. A 1.5-mm guide needle was inserted into the vertebral body through the pedicle, to the appropriate depth defined by fluoroscopy. A biopsy sheath was inserted through the biopsy needle, to the anterior third of the vertebral body height. After another round of fluoroscopic control, a reamer was used to make a hole in the vertebra, through which a balloon was placed into to create a void. Then, the bone cement was delivered into the void. After the third X-ray examination, the sheath was removed. The incision was sutured and dressed up with aseptic bandages.

### Postoperative care

Pain and other vital signs were addressed. At the first day after the surgery, the patient was allowed to walk with a waist support. All the patients were discharged within postoperative 3 days and required to wear waist supports for 3 weeks and take routine anti-osteoporotic drugs continuously.

### Indexes

Recorded were indexes about times of fluoroscopy, operative duration, LOS, complications, pre-operative and pre-discharge VAS score, JOA score, and Cobb’s angle. The fluoroscopy was performed during incision positioning, puncture needle insertion, balloon expansion, and bone cement penetration in the control group. With the navigation of robotic arm, the fluoroscopy was not performed in the Tirobot group when the needle was being inserted.

Pre-operative VAS score and JOA score were collected before the use of pain-relievers. Cobb’s angle between the most tilted upper and lower end of the vertebra was measured on a sagittal-plane radiograph with a semi-automated digital measurement system by an experienced doctor. The position of the cement was used to assess the accuracy of the operation. When the cement was completely placed in the vertebral body, the operation was considered as correct. When the cement leaked out of the vertebral body, the operation was considered incorrect.

### Statistical analysis

SPSS 16.0 was used for statistical analysis. Measurement data were presented as mean ± standard deviations and compared with *t* test. The Mann-Whitney *U* test was used to compare continuous variables that were not normally distributed and presented as medians and interquartile ranges. Enumeration data were presented as percentages and compared with chi-squared test. *P* < 0.05 was considered as statistically significant.

## Results

### Demographic data

All the patients were DXA-diagnosed with osteoporosis. Operations were performed within 1 week after the fracture. Mild-trauma-caused compression fracture was found in the thoracic vertebra of 13 cases (43.33%) and the lumbar vertebra of 17 cases (56.67%) in the Tirobot group (14 males (46.67%) and 16 females (53.33%), ages 61–89 years, mean 69.50 (65.25, 75.25) years) and 12 cases (40%) and 18 cases (60%) in the control group (13 males (43.33%) and 17 females (56.67%), ages 61–86 years, mean 70 (65.75, 78.75) years). No difference was found in T scores based on DXA: − 3.6 ± 0.7 in the Tirobot group and − 3.5 ± 0.6 in control group (*P* = 0.796). There was no significant difference in sex (*P* = 0.795) and age (*P* = 0.625) between both groups.

### Fluoroscopic times, operative duration, and LOS

There was a significant difference in fluoroscopy times between both groups (Table 1). The control group underwent more times of radiographic evaluation than the Tirobot group (31.53 ± 5.72 versus 9.80 ± 1.74). Operative duration (*P* = 0.615) and LOS (*P* = 0.183) were not significantly different.

**Table 1:** Fluoroscopic frequency, operative duration, LOS in two groups

	Tirobot group (*n* = 30)	Control group (*n* = 30)	*P* value
Fluoscopic frequency (times)	9.80 ± 1.74	31.53 ± 5.72	< 0.001
Operative duration (min)	49.97 ± 9.86	48.27 ± 15.51	0.615
LOS (days)	1.7 ± 0.84	2.07 ± 1.23	0.183

*LOS* length of stay

### Postoperative complications

**Fig. 3:**
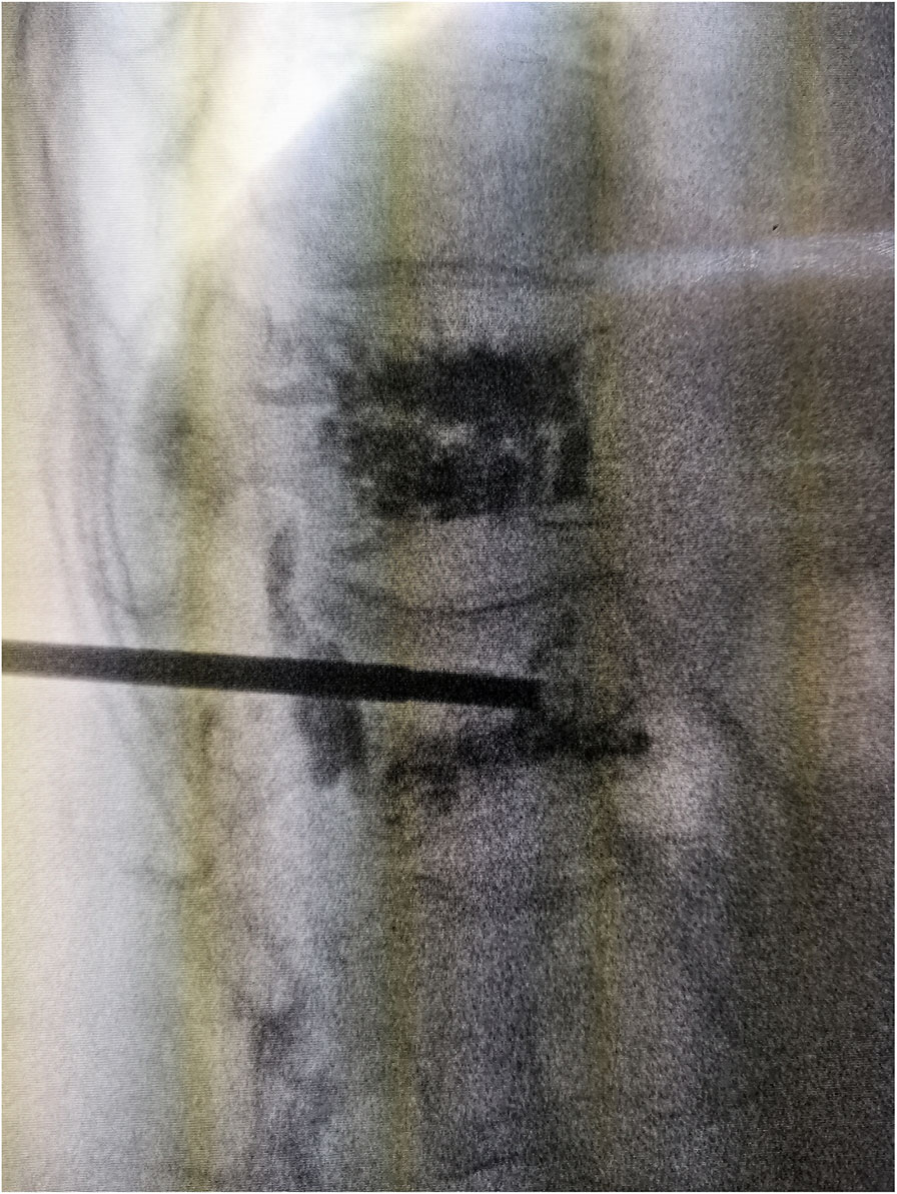
Cement leakage. The cement was visible on the posterior vertebral edge. The cement in the upper centrum had been injected 1 year before this surgical intervention

As shown in Table 2, bone cement leakage was found in seven cases (23%) in the control group (three of them developed neurological symptoms due to cement leakage) (Fig. 3) and none in Tirobot group. Two rates were different (*P* = 0.011). No other complications appeared, like infection and thrombosis.

**Table 2:** Postoperative complications in two groups

	Cement leakage	Leakage rate	Infection	Thrombosis
Tirobot group (*n* = 30)	0	0%	0	0
Control group (*n* = 30)	7	23%	0	0
*P* value	/	0.011	/	/

### Pre-operative and pre-discharge VAS score, JOA score, and Cobb’s angle

Pre-discharge VAS score was lower than pre-operative VAS score in both groups. Each group showed intra-group difference (*P* < 0.05), but both showed no between-group difference (*P* > 0.05) (Table 3). Pre-discharge JOA score was higher than pre-operative JOA score in both groups. Each group showed intra-group difference (*P* < 0.05), but both showed no between-group difference (*P* > 0.05) (Table 4). Pre-discharge Cobb’s angle was lower than pre-operative Cobb’s angle in both groups. Each group showed intra-group difference (*P* < 0.05), but both showed no between-group difference (*P* > 0.05) (Table 5).

**Table 3:** Pre-operative and pre-discharge VAS scores in two groups

	Pre-operative	Pre-discharge	***P*** value
Tirobot group (*n* = 30)	8.100 ± 1.062	2.700 ± 1.21	< 0.001
Control group (*n* = 30)	8.300 ± 0.915	3.067 ± 0.823	< 0.001
*P* value	0.438	0.175	

*VAS* visual analog scale

**Table 4:** Pre-operative and pre-discharge JOA scores in two groups

	Pre-operative	Pre-discharge	*P* value
Tirobot group (*n* = 30)	15.13 ± 2.50	23.33 ± 2.47	< 0.001
Control group (*n* = 30)	16.47 ± 3.16	23.97 ± 3.80	< 0.001
*P* value	0.075	0.448	

*JOA* Japanese orthopaedic association scores

**Table 5:** Pre-operative and pre-discharge Cobb’s angles in two groups

	Pre-operative	Pre-discharge	*P* value
Tirobot group (*n* = 30)	21.72 ± 2.85	10.54 ± 1.77	< 0.001
Control group (*n* = 30)	20.80 ± 3.17	10.87 ± 2.83	< 0.001
*P* value	0.244	0.585	

## Discussion

Thoracolumbar vertebral compression fracture is common in patients with osteoporosis, especially postmenopausal women, with an estimated number of 3,750,000 cases in 2020 [14, 15]. Previous studies have proved the superiority of vertebroplasty over conservative treatments [16–19]. However, in the former surgery, both patients and doctors face the damage from high radiation exposure, though protected by lead aprons, lead glasses, and lead gloves [6]. After 14 times of vertebroplasty, a surgeon may have received a radiation exposure that reaches the maximum one can endure during 1 year. However, a spine surgeon may accomplish more than 100 cases of vertebroplasty, indicating that excessive radiation exposure is common among spine surgeons [20, 21].

Compared to manual procedures, robot-assisted implantation brings with less radiation exposure, more precise location and better prognosis in vertebroplasty. Since 1992 in which a robotic system (Puma 260) was introduced into pedicle biopsy, robot-assisted vertebral surgeries have been revolutionized greatly [22]. Tirobot system, the third-generation orthopedic robot invented by Tian Wei [12], is being used at our hospital. With fluoroscopic guidance, the system displays high precision, good stability, minimal invasion, and reduced bleeding and radiation exposure [23, 24]. During the operation, the surgeon can visualize the deep structure and the robot can finish pre-designed procedures. Therefore, we evaluated the advantages of Tirobot in vertebroplasty.

Compared to bilateral vertebroplasty, unilateral vertebroplasty consumes less time and leaves less trauma [25, 26]. The reason that we only tested the efficacy of Tirobot in the later is that the procedures in this surgery, like pinpointing entry point of the needle, should be performed with high-precision biopsy and can be resolved by a robot. Besides, between the two modes of vertebroplasty, PKP has higher safety and better prognosis than percutaneous vertebroplasty (PVP) [27–29]. In clinical practice, PVP needs less time. Therefore, we applied Tirobot system in PKP, which is more suitable to test the usefulness of Tirobot in reducing operative duration.

Few reports about robot-assisted vertebroplasty have been published, indicating the difficulty in widening its application. In the operation, the tracer of the robot must be fixed, and especially during the implantation, the tracer must be tightly fastened to the spinous process. To realize this, however, the process must be totally exposed in prior, through which the skin and muscles have to be cut apart. Therefore, robot-assisted vertebroplasty brings more surgical wounds, thus greatly restricting its wide use. To solve this problem, we improved the Tirobot by introducing a base fixed with 1 to 3 K-wires, thereby avoiding the additional surgical incision. In using the new Tirobot system, the base of the tracer was stable and simple and the operation was less laborious (Fig. 1). Our technical innovation paves the way to the wide replication of robot-assisted vertebroplasty.

It is easy to fix the tracer to the lumbar process through vertically drilling with two K-wires (1.5 mm), but difficult when the process is narrow. We have tested the thickness of the processes in different vertebrae. The thickness reaches its maximum in T1 and minimum in T7: T1>T12>T2>T11>T10>T3>T9>T4>T5>T8>T6>T7. The processes of T3-T9 are all < 5 mm in thickness. Clinically, a process < 5 mm in thickness is hard to be fixed with the K-wire. Therefore, in fixing the tracer to T3-T9, we did not drill into the process from a direction vertical to its surface, but from a bevel direction (sometime supported by another K-wire). With this method, all the tracer bases were implanted in one setting, avoiding the use of fluoroscopy.

In this study, the Tirobot-assisted vertebroplasty achieved favorable outcomes, as shown by its high precision (precision < 1 mm) and safety [12]. All the biopsy procedures were accomplished in one setting. In the control group, however, the entry point and the needle direction had to be adjusted repeatedly under fluoroscopic guidance, thus increasing the operative duration and radiation exposure. Besides, in Tirobot-assisted vertebroplasty, the surgeon could make punctures from a wider range of angles. Consequently, the balloon could be well deflated, and cement injected into the right center of the vertebral body (Fig. 2). An ideal injection point can promote the distribution and reduce the leakage of bone cement and prevent the post-operative stress-induced fracture. In the present study, the results of cement leakage was not consistent with other studies [28, 30, 31]. This may be explained by the different evaluation criteria and the small size of clinical samples included in this study. We also found more favorable Cobb’s angle, complication rate, VAS, and JOA in Tirobot-assisted vertebroplasty. A shorter LOS also indicated the possibility of enhanced recovery after surgery (ERAS).

Limitations also exist in this study. First, the follow-up is short; long-term clinical observation is needed to evaluate the efficacy of Tirobot-assisted vertebroplasty. Second, the sample size is small. The insignificant difference in LOS and operative duration may arise from the scarcity of the samples. In future studies, we will prolong the follow-up time and increase the sample size to make the results more convincing. Supplementary explanation has been added in the article.

## Conclusion

Tirobot-assisted vertebroplasty can reduce surgery-related trauma, post-operative complications, and patients’ and operators’ exposure to radiation. As a safe and effective strategy, this technique can realize the quick recovery from thoracolumbar osteoporotic compression fracture.

## Data Availability

All data generated during this study are included in this published article.

## Abbreviations

PKP: Percutaneous kyphoplasty
PVP: Percutaneous vertebroplasty
ERAS: Enhanced recovery after surgery
LOS: Length of stay
KMC: Kinetic Medical Co., Ltd
TM: Translational medicine
TKA: Total knee arthroplasty
THA: Total hip arthroplasty
